# Cross-cultural validation of the Chinese version of the EmPHasis-10 questionnaire in connective tissue disease patients with pulmonary arterial hypertension and its relationship with risk stratification

**DOI:** 10.1186/s12890-022-02056-1

**Published:** 2022-07-05

**Authors:** Yue Shi, Xingbei Dong, Xiaoyun Hu, Li Weng, Yongtai Liu, Jinzhi Lai, Zhuang Tian, Jiuliang Zhao, Mengtao Li, Jinmin Peng, Qian Wang, Xiaofeng Zeng

**Affiliations:** 1 Department of Rheumatology and Clinical Immunology, Chinese Academy of Medical Sciences & Peking Union Medical College, National Clinical Research Center for Dermatologic and Immunologic Diseases (NCRC-DID), Ministry of Science & Technology, State Key Laboratory of Complex Severe and Rare Diseases, Peking Union Medical College Hospital (PUMCH), Key Laboratory of Rheumatology and Clinical Immunology, Ministry of Education, Beijing, 100730 China; 2 Medical Intensive Care Unit, Peking Union Medical College Hospital, Peking Union Medical College and Chinese Academy of Medical Sciences, No 1. Shuaifuyuan, Dongcheng District, Beijing, 100730 China; 3 Department of Cardiology, Peking Union Medical College Hospital, Peking Union Medical College and Chinese Academy of Medical Sciences, No 1. Shuaifuyuan, Dongcheng District, Beijing, 100730 China

**Keywords:** Connective tissue disease, Pulmonary arterial hypertension, Quality of life, Risk stratification

## Abstract

**Backgrounds:**

The EmPHasis-10 questionnaire is a disease-specific quality of life (QoL) measurement in patients with pulmonary hypertension. We report the results of cross-cultural validation of the Chinese version of the EmPHasis-10 and its relationship with risk stratification in patients with connective tissue disease-associated pulmonary arterial hypertension (CTD-PAH).

**Methods:**

The Emphasis-10 was administered to 75 CTD-PAH patients along with the 36-item Medical Outcomes Study Short Form Survey (SF-36) and EuroQol five dimensions questionnaire (EQ-5D). The diagnosis of PAH was confirmed by right heart catheterization. Demographic and clinical data were obtained. Multivariable logistic regression was conducted based on the low risk profile assessed by a 4-strata risk assessment model (COMPERA 2.0) at follow-up.

**Results:**

Date from 75 patients with CTD-PAH were analysed. The EmPHasis-10 demonstrated satisfactory reliability (Cronbach α = 0.95) and convergent validity showed by the significant relationship with WHO Functional Class (*P* = 0.003), SF-36 (*P* < 0.001) and EQ-5D (P = 0.002). EmPHasis-10 was significantly associated with achieving the low risk profile at 12 months of follow-up (Odds ratio: 0.928, *P* = 0.029) after adjusting for WHO Functional Class.

**Conclusion:**

EmPHasis-10 has acceptable reliability and validity in CTD-PAH patients and may serve as an additional parameter in risk stratification.

## Background

Pulmonary arterial hypertension (PAH) is a progressive disease of the pulmonary vasculature with high morbidity and mortality rate [1]. PAH is a severe complication of connective tissue disease (CTD) including systemic sclerosis (SSc), systemic lupus erythematosus (SLE), undifferentiated CTD (UCTD) and to a lesser extent, Sjögren’s syndrome (SS), rheumatoid arthritis (RA) and dermatomyositis [2]. PAH patients often present with dyspnea and functional limitations, negatively affecting patient’s health related quality of life (HRQoL) [3, 4]. Several generic instruments have been validated to assess HRQoL in PAH [5], but they lack some items relevant to PAH and may not always accurately capture the changes in HRQOL during the treatment. Currently more disease-specific HRQoL measurements have been designed for PAH patients, which appear to be important clinical end points and predictors of prognosis in PAH patients [6, 7]. EmPHasis-10 is a short, simple and powerful tool for assessing HRQoL in pulmonary hypertension patients [8]. It was suggested as an independent predictor of clinical outcome and has utility in risk assessment in addition to the parameters currently used [9].

A multiparametric stratification system has been recommended by the 2015 European Society of Cardiology (ESC) and the European Respiratory Society (ERS) pulmonary hypertension (PH) guidelines to assess response to therapy and predict mortality in PAH patients [10]. The prognostic value of risk stratification at baseline and follow-ups has been validated in several PAH cohorts [10] and also confirmed in CTD-PAH [11]. Furthermore, a recent study by Min et al. showed that higher risk stratification was associated with worse HRQoL in PAH patients [12]. A 4-strata risk assessment model based on three non-invasive criteria was confirmed by the Comparative, Prospective Registry of Newly Initiated Therapies for Pulmonary Hypertension (COMPERA) investigators and was proved to be sensitive to prognostically relevant changes in risk [13]. However, the relationships between EmPHasis-10 score and risk stratification in short- and long-term follow up have not been studied in CTD-PAH.

The haemodynamic parameters measured by right heart catheterization are essential for the diagnosis, disease assessment and therapy adjustment in CTD-PAH patients. However, inconsistent results have been reported about the impact of the revised PAH diagnostic criteria of haemodynamic parameters on clinical outcomes in CTD-PAH patients [14, 15]. In contrast, little information available on the association between haemodynamic characteristic, HRQoL and risk stratification. The aim of this study was to validate the reliability of EmPHasis-10 scale and further assess the relationship between quality of life (QoL) and risk stratification in CTD-PAH patients.

## Methods

### Study design, participants and data collection

This cross-sectional study included 75 incident and prevalent patients with PAH associated with CTD who were followed up and underwent right heart catheterization (RHC) at the Department of Rheumatology, Peking Union Medical College Hospital between January 2019 and January 2020. PAH is defined as the concomitant presence of mPAP ≥25 mmHg, PAWP ≤15 mmHg and PVR > 3 WU on RHC at sea level in a resting state [16]. Patients with an apparent pulmonary embolism confirmed by ventilation perfusion scintigraphy or computed tomographic pulmonary angiography; with moderate- to- severe ILD revealed by high- resolution CT scans or a pulmonary function test; or other causes of PAH confirmed by medical history inquiry, laboratory tests, and imaging were excluded. Demographic and clinical data were collected. Eligible patients were invited to complete the questionnaires including EmPHasis-10 and two generic QoL measurements: 36-item Medical Outcomes Study Short Form Survey (SF-36) and EuroQol five dimensions questionnaire (EQ-5D). We calculated risk at the follow-up visit in 6 and 12 months based on the COMPERA 2.0 risk assessment model using three noninvasive low-risk criteria: (1) World Health Organization Functional Class (WHO-FC) I or II; (2) six-minute walk distance (6MWD) > 440 m; (3) brain natriuretic peptide (BNP) < 50 ng·L^−1^ or N-terminal pro-brain natriuretic peptide (NT-proBNP) < 300 ng·L^−1^ [13].

EmPHasis-10 is a PAH-specific QoL measurement with 10 items [17]. Items are rated on a semantic 6-point scale (from 0 to 5), with lower scores indicating better quality of life. A Chinese translated version of EmPHasis-10 were used. The translation was approved by the original English developers and carried out using a forward–backward method by a health professional and an independent certified medical translator.

SF-36 is a validated generic measure of QoL and is widely used in PAH patients [5]. SF-36 is summarized in eight domains [physical functioning (PF), role physical (RP), bodily pain (BP), general health (GH), vitality (VT), social functioning (SF), role emotional (RE) and mental health (MH)] which can be combined into a PCS (physical component summary) and a MCS (mental component summary) score [18]. EQ-5D is another commonly used generic measure of health utility [19]. EQ-5D contains 5 items measuring patient mobility, self-care, usual activities, pain and anxiety/depression. Each dimension has three levels of response and the results are reported as a single utility index score between 0 and 1.

This study was approved by the Medical Ethics Committee of Peking Union Medical College Hospital and all patients provided written informed consent.

### Statistical analysis

Data were analyzed using R software (www.r-project.org). The fitness of data to normal distribution was tested by Shapiro–Wilk test. Continuous data were presented as mean ± SD or median (Interquartile range 25–75%) and categorical data were presented as number and percentage. Cronbach alpha coefficient was used to test the reliability of EmPHasis-10. Pearson or spearman correlation analysis was used to determine the relationship between EmPHasis-10 and the two generic QoL scales to obtain the convergent construct validity of EmPHasis-10. Association between WHO-FC and QoL outcomes were calculated using ANOVA. Univariable and multivariable logistic regression was performed using the enter method to analyze the factors associated with the low risk profile in CTD-PAH patients. EmPHasis-10 and parameters of known prognostic significance in PAH were utilized: age, WHO-FC, mean right atrial pressure (mRAP) and cardiac index (CI). *P* < 0.05 was considered statistically significant and all tests were two tailed.

## Results

### Baseline characteristics of patients with CTD-PAH

Table 1 shows characteristic of the 75 CTD-PAH patients enrolled in the QoL assessments. 98.7% of patients were female and the median duration of PAH was 5 years. The most common underlying CTD was SLE (56.7%), followed by SS (15.0%) and SSc (11.8%). The majority of patients (60.9%) were in WHO FC II. Mean right atrial pressure was 8.5 mmHg. All the patients (100%) received glucocorticoids, and 98.7% of them received immunosuppressive treatment. At least one PAH-specific treatment was administrated to 83.3% of patients, 54.2% of whom received endothelin receptor antagonist (ERA), 55.9% of whom received phosphodiesterase inhibitor (PDE5-I) and 3.4% of whom received prostacyclin analogue (PG). 16.3% patients received combination therapy consisting of two PAH targeted drugs.

**Table 1:** Patient baseline characteristic

Characteristic	Values (N = 75)
Female, %	98.7
Age, years	36.4 ± 11.9
Time since initial PAH diagnosis, months	49.5 (39.3–69.7)
BMI, kg/m^2^	22.4 ± 3.4
Education	
Middle school, %	23.2
High school, %	15.9
College, %	60.9
Marital status	
Married, %	74.3
Unmarried, %	23.1
Divorce,%	2.6
Smoking	
Yes, %	1.9
No, %	98.1
CTD etiology	
SLE, %	56.7
SS, %	15.0
SSc, %	11.8
UCTD, %	8.3
RA, %	1.7
Clinical features	
Arthritis, %	30.7
ILD, %	10.7
WHO functional class	
I, %	14.9
II, %	60.9
III, %	24.3
NT-proBNP, pg/ml	95.0 (39.0–390.0)
Echocardiography	
PASP, mmHg	55.9 ± 24.5
TAPSE, mm	18.9 ± 3.7
RV diameter, mm	40.9 ± 6.5
RV internal dimension, mm	24.6 ± 6.7
Pericardial effusion, %	17.7
LVEF, %	68.3 ± 6.9
RHC	
mRAP, mmHg	8.5 ± 2.9
mPAP, mmHg	38.9 ± 13.7
PAWP, mmHg	11.9 ± 3.6
PVR, WU	4.8 ± 3.1
CI, L/(min × m^2^)	3.7 ± 0.8
Treatment	
Glucocoticoid	
High-dose (≥ 40 mg/day), %	22.7
Medium-dose (15 mg/day < x < 40 mg/day), %	13.3
Low-dose (≤ 15 mg/day), %	64
Immunosuppressant	
CYC, %	30.5
MMF, %	30.5
TAC, %	40.7
HCQ, %	69.5
≥ 2, %	78.0
PAH medication	
ERA, %	54.2
PDE5-I, %	55.9
PG, %	3.4
≥ 2, %	16.3
Diuretic, %	66.1
Digoxin, %	37.3

Date expressed as mean ± standard deviation or median (Interquartile range:25%-75%)

WHO, World Health Organization; SLE, systemic lupus erythematosus; SS, sjogren syndrome; SSc, systemic sclerosis; UCTD, undifferentiated connective tissue diseases; RA, rheumatoid arthritis; ILD, Interstitial lung disease; NT-proBNP, N-terminal pro-brain natriuretic peptide; PASP, pulmonary arterial systolic pressure; TAPSE, tricuspid annular plane systolic excusion; RV, right ventricular; LVEF, left ventricular ejection fraction; RHC, right heart catheterization; mRAP, mean right atrial pressure; mPAP, mean pulmonary arterial pressure; PAWP, pulmonary arterial wedge pressure; PVR, pulmonary vascular resistance; CI, cardiac index; CYC, cyclophosphamide; MMF, mycophenolate mofetil; TAC. tacrolimus; HCQ, hydroxychloroquine; ERA, endothelin receptor antagonist; PDE5-I, phosphodiesterase inhibitor; PG, prostacyclin analogue

### Validity and reliability of EmPHasis-10

Criterion validity is suggested by strong correlation of EmPHasis-10 with WHO FC (Table 2). Patients with higher WHO-FC had significantly worse life quality (*P* = 0.003) measured by EmPHasis-10. There were also significant correlations between WHO-FC and QoL assessed by EQ-5D (*P* = 0.002) and some domains of SF-36 including physical functioning (*P* < 0.001), physical role functioning (*P* = 0.002) and emotional role functioning (*P* = 0.002). Moreover, these QoL measurements are strongly correlated (Table 3). The scores of EmPHasis-10 were strongly associated with PCS (*r* = −0.85, *P* < 0.001) and MCS (*r* = −0.81, *P* < 0.001) of SF-36 scale. Moderate correlation (*r* = 0.46, *P* < 0.001) was seen between EmPHasis-10 and PVR. Internal consistency reliability of EmPHasis-10 was found to be high for the Cronbach alpha value (α = 0.95).

**Table 2:** The relationship of the quality of life scores with WHO FC

	WHO-FC I (n = 11)	WHO-FC II (n = 45)	WHO-FC III (n = 18)	*P* value
EmPHasis-10	2.00 (0.00, 5.75)	10.50 (7.00, 22.25)	18.24 ± 11.48	0.003
SF-36				
Vitality	76.50 ± 17.17	63.84 ± 19.57	51.47 ± 25.54	0.019
Physical functioning	89.55 ± 9.07	80.00 (73.75, 90.00)	55.56 ± 22.55	< 0.001
Bodily pain	100.00 (100.00, 100.00)	74.00 (57.00,100.00)	69.69 ± 25.86	0.102
General health perceptions	60.40 ± 25.96	46.74 ± 19.64	39.00 ± 29.15	0.079
Physical role functioning	100.00 (100.00, 100.00)	50.00 (0.00,100.00)	25.00 (0.00,25.00)	0.002
Emotional role functioning	100.00 (100.00, 100.00)	66.67 (33.33,100.00)	33.33 (0.00,66.67)	0.002
Social role functioning	100.00 (75.00, 100.00)	75.00 (50.00,87.50)	58.33 ± 26.83	0.035
Mental health	84.00 ± 10.83	72.00 (58.00,88.00)	61.88 ± 22.05	0.023
PCS	86.75 (84.00, 94.25)	61.00 (42.25,82.75)	45.85 ± 22.79	< 0.001
MCS	91.00 (77.13, 92.31)	73.92 (49.50,85.13)	51.24 ± 28.80	0.004
EQ-5D	1.00 (1.00, 1.00)	0.94 (0.86, 1.00)	0.87 ± 0.10	0.002

Date expressed as mean ± standard deviation or median (Interquartile: 25–75%).

WHO-FC, World Health Organization Functional Class; SF-36, 36-item Medical Outcomes Study Short Form Survey; MCS, Mental Component Summary; PCS, Physical Component Summary; EQ-5D, EuroQol five dimensions questionnaire

**Table 3:** Correlations between quality of life questionnaires

	EmPHasis-10	SF-36		EQ-5D	mRAP	CI	PVR
		PCS	MCS		(mmHg)	(L/min/m^2^)	(WU)
EmPHasis-10		−0.85***	−0.82***	−0.72***	0.10	−0.20	0.46***
SF-36							
PCS	−0.85***		0.87***	0.76***	−0.15	0.22	−0.39***
MCS	−0.82***	0.87***		0.75***	−0.19	0.15	−0.31*
EQ-5D	−0.72***	0.76***	0.75***		−0.18	0.17	−0.28*

Correlations assessed by Pearson or Spearman-Rank tests as appropriate.

SF-36, 36-item Medical Outcomes Study Short Form Survey; MCS, Mental Component Summary; PCS, Physical Component Summary; EQ-5D, EuroQol five dimensions questionnaire; mRAP, mean right atrial pressure; CI, cardiac index; PVR, pulmonary vascular resistance

*p < 0.05, **p < 0.01, ***p < 0.001

### Factors associated with meeting the low risk criteria in 6 and 12 months

During the follow-up visits, 75.9% and 81.8% of patients achieved low-risk stratum in 6 and 12 months, respectively. Two multivariate analysis models were developed to identify the factors associated with low risk profile in 6 and 12 months (Table 4). Age, WHO-FC, mRAP, CI and EmPHasis-10 were added into models. EmPHasis-10 was an independent predictor of meeting the low risk criteria after 12 months (Odds ratio: 0.928, 95% confidence interval: 0.868–0.993, *P* = 0.029).

**Table 4:** Univariable and multivariable logistic regression analysis of achieving the low risk profile in 6 and 12 months

	Low risk in 6 months						Low risk in 12 months					
	Univariable			Multivariable			Univariable			Multivariable		
	OR	95% CI	*P* value	OR	95% CI	*P* value	OR	95% CI	*P* value	OR	95% CI	*P* value
Age	0.980	(0.939, 1.023)	0.359	0.985	(0.938, 1.035)	0.557	1.013	(0.958, 1.071)	0.650	1.044	(0.973, 1.121)	0.228
WHO FC III (ref I&II)	0.519	(0.130, 2.078)	0.354	0.279	(0.938, 1.035)	0.279	0.805	(0.217, 2.991)	0.746	0.612	(0.101, 3.721)	0.594
mRAP, mmHg	0.996	(0.827, 1.199)	0.965	1.022	(0.817, 1.278)	0.851	1.037	(0.843, 1.276)	0.732	1.111	(0.865, 1.428)	0.410
CI	0.592	(0.306, 1.143)	0.118	0.549	(0.252, 1.195)	0.131	0.868	(0.430, 1.753)	0.693	0.636	(0.273, 1.482)	0.294
EmPHasis-10	0.984	(0.935, 1.034)	0.521	0.973	(0.921, 1.029)	0.335	0.941	(0.890, 0.995)	0.031*	0.928	(0.868, 0.993)	0.029*

WHO-FC, World Health Organization Functional Class; mRAP, mean right atrial pressure; CI, cardiac index

*p < 0.05, **p < 0.01, ***p < 0.001

## Discussion

This is the first study set out with the aim of validating the Chinese version of EmPHasis-10 and assessing the impact of patient-reported outcome on risk stratification in CTD-PAH patients. This study indicate that EmPHasis-10 is a reliable and valid questionnaire measuring QoL in CTD-PAH patients. Another important finding was that EmPHasis-10 was identified as an associated factors of risk stratification at 12-month follow-up when adjusting for haemodynamics and WHO FC.

The Chinese version of EmPHasis-10 has shown good validity and sensitivity in assessing QoL in CTD-PAH patients. Functional parameters such as WHO FC in PAH patients are strongly correlated with self-assessment of QoL [6, 20], which is consistent with our finding. EmPHasis-10 also demonstrated significant correlation with the generic QoL instruments. These findings support good convergent validity of EmPHasis-10. A new haemodynamic definition of PAH was proposed by the 6th Pulmonary Hypertension World Symposium, suggesting mean pulmonary arterial pressure (mPAP) > 20 mmHg as above the upper limit of normal in combination with pulmonary arterial wedge pressure (PAWP) ≤ 15 mm Hg and pulmonary vascular resistance (PVR) ≥ 3 WU measured by right heart catheterization [16]. This new definition has raised more attention for the relationship between haemodynamic parameters and clinical outcomes [14, 15, 21]. However, only moderate correlation was seen between EmPHasis-10 and PVR. Lewis et al. also reported only weak correlations between this instrument and pulmonary haemodynamics [22]. This highlights the potential role of EmPHasis-10 as a quantitative measure of patient's functional ability and as an additional therapeutic target from the patients' point of view.

EmPHasis-10 is a PAH-specific QoL scale containing items that cover breathlessness, energy, social confidence and independence [17]. In previous clinical trials in CTD-PAH, some generic QoL measurements were often used and the overall changes of QoL made by different therapeutic intervention were hard to detect [23, 24]. Our findings showed that EmPHasis-10 could be used as a disease-specific assessment of QoL in CTD-PAH and was associated with risk stratification at follow-up. Hence, more disease-specific instruments like EmPHasis-10 should be used in a complementary fashion when assessing CTD-PAH patients. Nevertheless, musculoskeletal involvement in CTD-PAH patients (especially in SSc and RA) could also have a major impact on HRQoL and inflammation also plays an important role in SLE associated PAH [25], which should be taken into account in the HRQoL evaluation. Thus, the adoption of disease-specific QoL measurements into different type of PAH need to be study further. Some cardiopulmonary exercise programs were developed in patients with PAH, showing a significant improvement of QoL [26]. EmPHasis-10 would be a suitable evaluation tool to quantify the change of QoL in these exercise training programs.

The recommended risk stratification strategy has proved successful in many PAH registries [27–30]. A UK multi-center study have demonstrated that EmPHasis-10 could be used in an exploratory risk stratification approach to identify distinct risk groups with significant one-year motality [22]. The short-term outcome used in our studies was low risk COMPERA 2.0 score proposed by the COMPERA Registry investigators [13]. Due to reasons including the invasive nature and availability, hemodynamic assessment was not included in every follow-up assessment. Thus, the COMPERA 2.0 model is based on three non-invasive parameters that have been thoroughly validated in previous studies. This model was proved to be a useful tool for determining prognosis for pulmonary hypertension patients in several registries [13, 31]. In our study, EmPHasis-10 was identified as an associated factors of low risk stratum in 12 months. Due to the prognostic value of QoL assessments, our study supports the possibility of adding QoL into currently used parameters in risk stratification. More work is needed to determine the precise stratified criteria.

Our study has several limitations: (1) the information about 6-min walk distance was not fully recorded, which may cause biases in our results; (2) the sample size was relatively small for the subgroup analyses. Further studies are needed to assess the relationship between EmPhasis-10 and changes in risk stratification during follow-ups. (3) most of our patients were WHO FC I, II or III, so our findings could not used in patients with more severe functional status. (4) the major underlying cause of CTD-associated PAH in our study is SLE and the mean age is relatively lower, which are consistent with our previous retrospective studies [32]. This may reduce the applicability of our results. (5) Further studies are needed to assess the relationship between repeated EmPHasis-10 scores in the follow-up and risk stratification.

## Conclusion

In conclusion, EmPHasis-10 is a suitable instrument for measuring HRQoL in CTD-PAH and an independent predictor of risk stratification at 12-month follow-up. HRQoL may serve as an additional parameter in the risk stratification tool in order to improve the self-reported outcomes of CTD-PAH patients.

## Data Availability

The datasets used and analysed during the current study are available from the corresponding author on reasonable request.

## Abbreviations

CTD-PAH: Connective Tissue Disease-associated Pulmonary Arterial Hypertension
SF-36: The 36-item Medical Outcomes Study Form Survey
EQ-5D: EuroQol five dimensions questionnaire
WHO-FC: World Health Organization Functional Class
SSc: Systemic sclerosis
SLE: Systemic lupus erythematosus
UCTD: Undifferentiated CTD
SS: Sjogren syndrome
RA: Rheumatoid arthritis
HRQoL: Health related quality of life
ESC: The European Society of Cardiology
ERS: The European Respiratory Society
PH: Pulmonary hypertension
mPAP: Mean pulmonary arterial pressure
PAWP: Pulmonary arterial wedge pressure
PVR: Pulmonary vascular resistance
QoL: Quality of life
BNP: Brain natriuretic peptide
NT-proBNP: N-terminal pro-brain natriuretic peptide
ECHO: Echocardiography
PF: Physical functioning
RP: Role physical
BP: Bodily pain
GH: General health
VT: Vitality
SF: Social functioning
RE: Role emotional
MH: Mental health
PCS: Physical component summary
MCS: Mental component summary
mRAP: Mean right atrial pressure
ERA: Endothelin receptor antagonist
PDE5-I: Phosphodiesterase inhibitor
PG: Prostacyclin analogue
ILD: Interstitial lung disease
PASP: Pulmonary arterial systolic pressure
TAPSE: Tricuspid annular plane systolic excusion
RV: Right ventricular
LVEF: Left ventricular ejection fraction
RHC: Right heart catheterization
CI: Cardiac index
CYC: Cyclophosphamide
MMF: Mycophenolate mofetil
TAC: Tacrolimus
HCQ: Hydroxychloroquine
